# Consumption of red and processed meat during early pregnancy and risk of gestational diabetes: a prospective birth cohort study

**DOI:** 10.1038/s41598-024-55739-6

**Published:** 2024-03-03

**Authors:** Reyhane Norouziasl, Ahmad Jayedi, Majid Mirmohammadkhani, Alireza Emadi, Shahrzad Aghaamo, Sakineh Shab-Bidar

**Affiliations:** 1 Student Research Committee, Semnan University of Medical Sciences, Semnan, Iran; 2 Social Determinants of Health Research Center, Semnan University of Medical Sciences, Semnan, Iran; 3 Food Safety Research Center (Salt), Semnan University of Medical Sciences, Semnan, Iran; 4 Research Center of Abnormal Uterine Bleeding, Semnan University of Medical Sciences, Semnan, Iran; 5 Department of Community Nutrition, School of Nutritional Science and Dietetics, Tehran University of Medical Sciences, No 44, Hojjat-Dost Alley, Naderi St., Keshavarz Blvd, P. O. Box 14155/6117, Tehran, Iran

**Keywords:** Metabolic disorders, Nutrition disorders

## Abstract

To investigate the association of red and processed meat intake with the risk of gestational diabetes (GDM) in Iranian mothers. A total of 635 pregnant mothers were included. Dietary intake was assessed by a 90-item food frequency questionnaire during the first trimester of pregnancy. Intakes of total red meat, unprocessed red meat, and processed meat were calculated and then, Cox proportional hazard model was used to calculate the hazard ratios (HR) and 95%CIs of GDM across tertiles of red meat intake while controlling for age, occupation, pre-pregnancy body mass index, physical activities, history of cardiovascular disease, hypertension, hypothyroidism, hyperthyroidism, and pregnancy hypertension, order of pregnancy, nausea during current pregnancy, multivitamin use during current pregnancy, weight gain during current pregnancy and total energy intake. The average age of the mothers was 28.80 ± 5.09 years, the average pre-pregnancy body mass index was 25.13 ± 4.43 kg/m^2^, and the average weight gain during pregnancy was 13.50 ± 5.03 kg. The multivariable-adjusted HRs of GDM for the third tertiles of red and processed meat, red meat, and processed meat intake were, respectively, 1.92 (95% CI 1.06, 3.49), 1.52 (95% CI 0.85, 2.72) and 1.31 (95% CI 0.73, 2.34) when compared to the first tertiles. Our prospective cohort study suggested that there was a positive association between the consumption of red and processed meat and with risk of GDM in a small sample of Iranian mothers with low red meat intake. More large-scale cohort studies in the Iranian population are needed to present more robust evidence in this regard.

## Introduction

Gestational diabetes mellitus (GDM), a common complication during pregnancy, is characterized by the initial manifestation or first appearance of impaired glucose tolerance during pregnancy^1^. Compared to the global standardized prevalence of GDM, which is 14%^2^, this rate ranges from 1.3 to 18.8% in Iranian pregnant women^3^. GDM is linked with unfavorable outcomes such as neonatal macrosomia and hypoglycemia^4^. The occurrence of Type 2 diabetes is more likely among women who have previously experienced GDM, thereby emphasizing the necessity of identifying modifiable risk factors to diminish the likelihood of developing GDM^5^.

Evidence points to a connection between eating choices before and during pregnancy and the risk of GDM^6^. A high intake of red and processed meat, along with increased adherence to a Western dietary pattern, may potentially be associated with an elevated susceptibility to developing GDM^7^. Unhealthy cooking methods such as frying in ketchup, cooking with fried vegetables, and grilling may increase the production of dangerous substances such as advanced glycation end products, polycyclic aromatic hydrocarbons (PAHs), and heterocyclic amines (HCAs)^8^. These products can interfere with insulin sensitivity and, thus, may be linked to a higher risk of type 2 diabetes^9^.

However, studies investigating the association of red meat with the risk of GDM are controversial. An increased intake of red meat was found to be positively associated with the risk of GDM in a prospective observational study conducted in India^10^. In contrast, a cohort study in Singapore that looked at the relationship between red meat protein consumption and the risk of GDM did not report any significant association^11^. Although there are several investigations on the association between dietary patterns and the risk of GDM in Iran^12–14^, studies of the association between the consumption of red and processed meat and the risk of GDM in the Iranian population are lacking. In general, Iranians consume less red and processed meat than those living in Western countries. Iranian traditional diet is rich in carbohydrates and refined grains. Considering the recent controversy on the link between the consumption of red and processed meat and multiple health outcomes^15,16^, it is uncertain whether the consumption of red and processed meat in a population with low red meat consumption is associated with the risk of developing GDM. Hence, the present prospective cohort study aimed to assess the association of the consumption of red and processed meat during early pregnancy with the risk of GDM in a sample of Iranian women.

## Materials and methods

### Participants

The present study was carried out within the framework of the Persian (Prospective Epidemiological Research Studies in IRAN) Birth Cohort^17^. The Persian Birth Cohort is an ongoing, national, prospective cohort research in five districts in Iran to advance knowledge and supply scientific proof for the creation of evidence-based national policies on various developmental causes of health and disease^17^. Pregnant women in Semnan, a city in central Iran, were chosen as participants. Pregnant women in Semnan who were referred to medical facilities between 2018 and 2020 were asked to take part in this prospective cohort research. Additionally, to entice women to join in this prospective cohort research, we distributed advertising through local and social media, as well as at medical clinics located around the city. Women of Iranian descent who are in their first trimester of pregnancy, regardless of gravidity, parity, or use of fertility therapy, who have lived in Semnan for at least a year, and who want to give birth in a hospital in Semnan met the inclusion criteria. Pregnancies that ended in a cesarean section or a normal vaginal birth were both included. Twin pregnancies, disorders connected to hormones, and hormone treatment were excluded criteria.

1024 women in total consented to take part in the study. For the present study, a total of 635 pregnant women were included after excluding certain groups of individuals. Including, mothers with insufficient information on dietary intake (n = 281), those who had incomplete information about study outcomes and did not continue until the end (n = 45), mothers with outlier energy intake (n = 18), those who smoked cigarettes (n = 10), and mothers with a previous history of GDM (n = 35) were excluded from the analyses. The study's procedure was described to each participant, who also completed an informed consent form. The ethics committee of the Semnan University of Medical Sciences (Ethic code: IR.SEMUMS.REC.1401.233) approved the study protocol.

### Assessment of dietary intake

A food frequency questionnaire consisting of 90 items that were created and validated for use in this prospective cohort research was used to assess the individuals' dietary intake throughout the first trimester of pregnancy^17^. Dietary evaluations were carried out by professional interviewers conducting in-person interviews. We asked mothers to indicate how frequently they consumed the food items included in the food frequency questionnaire throughout their first trimester of pregnancy. There were nine multiple-choice options for frequency responses, ranging from "never or less than once a month" to "6 or more times per day" depending on the type of food item. Every stated consumption frequency was converted to grams per day using standard units. To determine the total calorie and nutrient intakes, Nutritionist IV software (version 7.0; N-Squared Computing, Salem, OR), customized for Iranian meals, was used.

### Outcome assessment

We followed the American Diabetes Association criteria for diagnosis of GDM^18^. Accordingly, mothers who had at least two of the following criteria were considered as having GDM: fasting plasma glucose higher than 95 mg/dL, 1-h plasma glucose higher than 180 mg/dL, 2-h plasma glucose higher than 155 mg/dL, and 3-h plasma glucose greater than 140 mg/dL.

### Assessment of other variables

Using organized, pre-tested questionnaires created for use in Persian Birth Cohorts, trained interviewers gathered information about the research participants' characteristics^17^. Interviewers with training took down details about the subjects' ages, medical histories, educational levels, occupations of their mothers and fathers, and family income. We employed the internationally accepted and verified International Physical Activity Questionnaire (IPAQ) to assess the levels of physical activity^19^. The participants were categorized into two groups based on their Metabolic Equivalent minutes per week (MET-min/week) values, i.e., no or low physical activity (< 3000 MET-minute/week) and moderate and high physical activity (> 3000 MET-minute/week)^20^. An experienced interviewer took measurements of weight and height. Weight was assessed at the study's baseline using a digital scale, with light clothing and no shoes, to the closest 0.5 kg. In the second and third trimesters, weight measurements were repeated. Before birth, the mothers' final weights were assessed in the hospital using the same methodology. The difference between the first and last weights was used to compute weight gain. The participants were asked to stand without shoes with their shoulders touching the wall while having their height measured with a tab that was measured to the closest 0.5 cm. Based on the division of the weight in kilograms by the square of the height in meters, the body mass index (BMI) was derived.

### Plant or plant material

Our prospective cohort study was observational and we did not use any plant or plant material in this research.

### Statistical analyses

We first classified red and processed meat consumption into tertiles and then grouped study participants by tertiles of red and processed meat intake. Second, an analysis was conducted to compare participants' characteristics across tertiles of red and processed meat intake. We compared values of continuous and categorical variables between categories of red and processed meat intake using ANOVA and chi-squared tests, respectively. We calculated the hazard ratio (HR) and 95% confidence interval (CI) of GDM across categories of red and processed meat intake using the Cox proportional hazard model. Age, having a job with income, pre-pregnancy BMI, history of cardiovascular disease (yes/no), weight gain during current pregnancy (kg), history of hypertension, hypothyroidism, and pregnancy hypertension (yes/no), nausea during current pregnancy (yes/no), use of multivitamins during current pregnancy (yes/no), order of pregnancy, physical activities (no or low/moderate to high), and total energy intake were all taken into consideration for multivariable-adjusted analyses. SPSS (SPSS Inc., version 22) was used for all statistical analyses. P-values less than 0.05 were regarded as significant.

### Ethics approval and consent to participate

This study was conducted according to the guidelines laid down in the Declaration of Helsinki and all procedures involving research study participants were approved by the ethics committee of Semnan University of Medical Sciences. Written informed consent was obtained from all subjects/patients.

## Results

The characteristics of the study participants (n = 635) for each category of red and processed meat intake are displayed in Table 1. The average age of the mothers was 28.80 ± 5.09 years. The average pre-pregnancy BMI was 25.13 ± 4.43 kg/m^2^, and the average weight gain during pregnancy was 13.50 ± 5.03 kg.

**Table 1:** Characteristics of the study participants across tertiles of intake of red and processed meat (n = 635).

Variable^1^	Tertile 1 (n = 208)	Tertile 2 (n = 221)	Tertile 3 (n = 206)	P-value	P-trend	Comparison group^2^	Post hoc (P)
Age (years)	28.64 ± 5.04	28.81 5.28	28.79 4.79	0.929	0.665		
Prepregnancy BMI (kg/m^2^)	24.73 4.53	25.30 4.27	25.07 4.45	0.413	0.478		
Weight gain during current pregnancy (kg)	13.21 4.76	13.38 4.67	13.95 5.45	0.283	0.593		
Having job with income (%)				0.948			
Yes	60 (28.3)	58 (27.2)	60 (28.6)				
No	152 (71.7)	155 (72.8)	150 (71.4)				
University graduate (%)				< 0.001			
Yes	5 (2.3)	6 (2.6)	19 (8.9)			T1 VS Yes	.05743
No	211 (97.7)	222 (97.4)	195 (91.1)			T2 VS Yes	.08913
						T3 VS Yes	.00022
						T1 VS No	.05743
						T2 VS No	.08913
						T3 VS No	.00022
Physical activity				0.091			
Low (%)	172 (81.1)	157 (73.7)	153 (72.9)				
Moderate (%)	40 (18.9)	56 (23.3)	57 (27.1)				
History of CVD (%)				0.356			
Yes	1 (0.5)	2 (0.9)	4 (1.9)				
No	211 (99.5)	211 (99.1)	206 (98.1)				
History of hypertension (%)				0.304			
Yes	6 (2.8)	2 (0.9)	6 (2.9)				
No	206 (97.2)	211 (99.1)	204 (97.1)				
History of hypothyroidism (%)				0.563			
Yes	33 (15.6)	37 (17.4)	41 (19.5)				
No	179 (84.4)	176 (82.6)	169 (80.5)				
History of hyperthyroidism (%)				0.372			
Yes	2 (0.9)	4 (1.9)	4 (0.5)				
No	210 (99.1)	209 (98.1)	209 (99.5)				
History of pregnancy hypertension (%)							
Yes	0 (0)	0 (0)	0 (0)				
No	212 (100)	213 (10)	210 (100)				
Order of pregnancy (≥ 3, %)	44 (20.7)	54 (25.3)	25 (12)	0.028			
Nausea during current pregnancy (%)				0.21			
Yes	124 (58.5)	99 (46.5)	99 (47.5)				
No	88 (41.5)	114 (53.5)	111 (52.9)				
Multivitamin use during current pregnancy (%)				0.009			
Yes	15 (7.1)	21 (9.9)	34 (16.2)			T1 VS Yes	.54851
No	197 (92.9)	192 (90.1)	176 (83.8)			T2 VS Yes	.01242
						T3 VS Yes	.00270
						T1 VS No	.54851
						T2 VS No	.01242
						T3 VS No	.00270

P value less than 0.05 was considered significant.

One-way ANOVA for quantitative data and Chi-squared test for qualitative data have been used.

*BMI* body mass index, *CVD* cardiovascular diseases, *T* tertile.

^1^Values are mean ± standard deviation or the number (percentage).

^2^Post-hoc test for comparison between tertiles of red and processed meat intake and categories of general characteristics of participants.

Mothers who ate more red and processed meat were more likely to be university graduates and less likely to have nausea during current pregnancy. Moreover, mothers with the highest tertile of red and processed meat intake used more multivitamins during their current pregnancy. Other characteristics of the mothers did not differ significantly across tertiles of red and processed meat intake.

Intake of micro- and macronutrients and food groups across tertiles of red and processed meat intake are described in Table 2. In our study population, a higher intake of red and processed meat was accompanied by higher intakes of energy, carbohydrate, total fat, total protein, saturated fat, monounsaturated fatty acids, dietary fiber, magnesium, and calcium. In addition, Table 2 presents food group intakes based on tertiles of red and processed meat intake, where a higher intake of red and processed was related to a higher intake of fruit, beans and nuts, sweets, poultry, and egg.

**Table 2:** Dietary intake of the study participants across tertiles of intake of red and processed meat (n = 635).

Variable^1^	Tertile 1 (n = 208)	Tertile 2 (n = 221)	Tertile 3 (n = 206)	P-value^2^	Comparison group^3^	Post hoc (P)
Energy (kcal/d)	1829.34 ± 806.35	1681.61 ± 470.26	1798.35 ± 552.61	0.022	1 VS 2	0.001
					1 VS 3	< 0.001
					2 VS 3	0.076
Nutrients						
Carbohydrate (g/d)	245.98 ± 108.95	218.73 ± 61.32	235.22 ± 77.33	0.055		
Total fat (g/d)	67.52 ± 27.04	64.71 ± 18.96	70.18 ± 18.82	0.067		
Total protein (g/d)	53.42 ± 26.85	51.53 ± 17.72	60.30 ± 22.83	< 0.001		
					1 VS 2	< 0.001
					1 VS 3	< 0.001
					2 VS 3	< 0.001
Saturated fat (g/d)	19.14 ± 7.05	18.98 ± 5.90	20.33 ± 6.17	0.014		
PUFA (g/d)	21.15 ± 9.86	20.05 ± 7.38	21.01 ± 6.62	0.967		
MUFA (g/d)	15.09 ± 6.07	15.38 ± 4.41	17.18 ± 4.70	< 0.001		
					1 VS 2	< 0.001
					1 VS 3	< 0.001
					2 VS 3	< 0.001
Dietary fiber (g/d)	16.07 ± 7.56	13.94 ± 4.57	15.53 ± 6.61	0.033		
					1 VS 2	0.704
					1 VS 3	0.002
					2 VS 3	0.024
Vitamin C (mg/d)	280.56 ± 231.35	231.41 ± 98.27	255.09 ± 137.94	0.084		
Magnesium (mg/d)	266.28 ± 126.66	237.77 ± 71.73	260.82 ± 90.48	0.128		
Calcium (mg/d)	841.65 ± 489.64	782.35 ± 328.61	804.88 ± 372.84	0.499		
Food groups						
Whole grains (g/d)	24.20 ± 28.24	23.71 ± 23.08	20.00 ± 20.80	0.115		
Dairy (g/d)	371.04 ± 292.83	347.44 ± 213.88	350.00 ± 220.91	0.512		
Fruits (g/d)	401.43 ± 268.55	335.44 ± 192.81	402.09 ± 254.52	0.096		
Vegetables (g/d)	270.88 ± 152.30	248.14 ± 101.77	255.82 ± 127.99	0.658		
Beans and nuts (g/d)	16.15 ± 13.28	17.81 ± 9.39	19.21 ± 11.20	0.004		
					1 VS 2	< 0.001
					1 VS 3	< 0.001
					2 VS 3	0.333
Sweets (g/d)	259.36 ± 204.35	229.89 ± 143.85	244.71 ± 172.98	0.853		
Poultry (g/d)	8.63 ± 10.11	9.35 ± 5.80	11.29 ± 11.53	0.006		
					1 VS 2	0.017
					1 VS 3	< 0.001
					2 VS 3	0.191
Egg (g/d)	18.12 ± 17.30	22.38 ± 16.49	27.25 ± 30.10	< 0.001		
					1 VS 2	0.004
					1 VS 3	< 0.001
					2 VS 3	0.097

*BMI* body mass index, *MUFAs* monounsaturated fatty acids, *PUFAs* polyunsaturated fatty acids, *CVD* cardiovascular diseases.

^1^Values are mean ± standard deviation.

^2^ANCOVA was used to compare means after adjustment for age job status, pre-pregnancy BMI, history of CVD, hypertension, hypothyroidism, hyperthyroidism, pregnancy hypertension, order of pregnancy, nausea during current pregnancy, multivitamin use during current pregnancy, physical activities, weight gain during current pregnancy and total energy intake.

^3^Tertiles versus each one.

Table 3 indicates the HR and 95% CI of GDM (n = 80 cases) across tertiles of red and processed meat intake. In the multi-variable adjusted model, participants in the third tertile of red and processed meat intake had a higher risk of GDM compared to those in the first tertile (HR 1.92, 95% CI 1.06, 3.49). There was no association between red or processed meat intake and risk of GDM.

**Table 3:** Hazard ratio (95% CI) of gestational diabetes by tertiles of red and processed meat intake (n = 635).

Tertile	Tertile 1	Tertile 2	Tertile 3
Red and processed meat (g/d)	0–8.22	8.55–14.14	14.25–90
Participants//cases	212/17	213/34	210/29
Person-week	7882	7600	7699
Unadjusted HR and 95%CI	1.0	1.64 (0.90, 2.99)	2.13 (1.19, 3.82)
Multivariable-adjusted HR	1.0	1.37 (0.74, 2.52)	1.92 (1.06, 3.49)
P-value	–	0.319	0.032
Red meat (g/d)	0–4	4–13	13–90
Participants//cases	212/22	211/28	212/30
Person-week	8034	7992	7776
Unadjusted HR and 95%CI	1	1.21 (0.71, 2.09)	1.81 (1.04, 3.15)
Multivariable-adjusted HR	1	0.97 (0.56, 1.67)	1.52 (0.85, 2.72)
P-value	–	0.814	0.039
Processed meat (g/d)	0	0.1–2.9	3.3–31.1
Participants//cases	386/29	124/21	125/16
Person-week	14,590	4388	4203
Unadjusted HR and 95%CI	1	1.54 (0.92, 2.60)	1.16 (0.65, 2.05)
Multivariable adjusted HR	1	1.56 (0.92, 2.65)	1.31 (0.73, 2.34)
P-value	–	0.102	0.364

P value was obtained by cox regression analysis, adjusted for age, having job with income, pre-pregnancy body mass index, history of cardiovascular disease, hypertension, hypothyroidism, hyperthyroidism, pregnancy hypertension, order of pregnancy, nausea during current pregnancy, multivitamin use during current pregnancy, physical activities, weight gain during current pregnancy and total energy intake.

*HR* hazard ratio.

## Discussion

In this study, we looked into the relationship between a sample of Iranian women's risk of GDM and their consumption of red and processed meat during the early stages of pregnancy. Our prospective birth cohort study demonstrated that there was a positive link between the risk of GDM and the consumption of red and processed meat.

Evidence on the association of red and processed meat with the risk of GDM is conflicting. In accordance with our study, a prospective evaluation in the Nurses' Health Study II, which included 21,457 mothers with singleton pregnancies, found that mothers in the highest quintile of total red meat consumption had twice the risk of GDM than mothers in the lowest quintile^21^. Higher consumption of animal-based proteins, especially those from red meat, in the second trimester of pregnancy was highly related to the risk of developing GDM, according to a prospective cohort study of 452 pregnant women in Malaysia^22^. Furthermore, more red meat consumption during the second trimester of pregnancy was linked to an increased risk of GDM, according to a Chinese cohort study; however, there was no link between red meat consumption during the first trimester and that risk^23^.

In contrast to our results, there was no correlation between the consumption of red and processed meat and other animal-based dietary protein sources and the probability of GDM, according to a small hospital-based case–control research in Iran^24^. Another cohort study of 980 mothers with singleton pregnancies in Singapore suggested no connection between the consumption of red meat proteins and the risk of developing GDM^11^. A prospective cohort study on 1178 newly diagnosed cases of type 2 diabetes in Japan concluded that there was no significant relationship between the consumption of red and processed meat and the risk of type 2 diabetes in women^25^. The analysis of 1733 participants in Eastern Massachusetts in a prospective cohort study found no evidence that red or processed meats are associated with the risk of developing impaired glucose tolerance or GDM^26^.

The modulation of skeletal muscle glucose uptake and glycogen synthesis, hepatic glucose production, and insulin secretion provide evidence for the importance of amino acids in glucose homeostasis^27^. An observational study found that eating a high-protein diet for 6 months increased the amount of insulin released when glucose levels reached a certain level. This was because the endocrine -cells' glucose threshold was lowered, endogenous glucose output and plasma glucagon levels were increased, and gluconeogenesis was boosted^28^. Additionally, studies have shown that roasting or grilling red meat at high temperatures over an open flame can result in the production of various hazardous compounds, including heteroaromatic amines and PAHs^29^. Research have shown correlations between urine PAH biomarkers and inflammatory indicators such as 1- and 2-hydroxynaptol and 2-hydroxyphenanthrene, as well as a higher prevalence of type 2 diabetes^30,31^. Additionally, mounting evidence indicates that cooking beef at high temperatures may cause the development of advanced glycation end products, which have been related in both animal and human studies to oxidative stress, inflammation, and insulin resistance^32,33^.

In general, the average intake of red meat in Iranian population is lower than those living in Western countries. For instance, the Nurse's Health Study found that daily intake of total red meat varied from 20 to 150 g^21^, compared to 0 to 90 g/d in the present study. Our findings suggest that higher intake of red and processed meat, even in a population with such a low intake, may be positively associated with the risk of GDM. The average intake of red and processed meat in our study (12.90 g/day) was comparable to the previous hospital-based case–control study in Iran in 2019 (13.17 g/day)^24^. The inconsistency in the findings between our study and those found in the previous study in Iran may be due to a difference in study design (prospective cohort versus hospital-based case–control) and their small sample size (n = 320)^24^. In a previous publication from the Persian Birth Cohort, we found that greater adherence to the Mediterranean diet during early pregnancy was associated with a lower risk of GDM in Iranian mothers^34^. Evidence from previous research suggests that individual components of the Mediterranean dietary pattern are not equal in relation to prevention of cardiovascular disease^35^ and, thus, more research is needed to examine the effect of specific food groups included in the Mediterranean diet score. Our findings in the present study suggest that higher intake of red and processed meat, even in a population with such a low red meat intake, may be associated with a higher risk of GDM. A recent evaluation within the EPIC-InterAct Study indicated that replacement of red and processed meat with other dietary protein sources including cheese, yogurt, nuts, and cereals was associated with a lower risk of type 2 diabetes^36^. An evaluation within the Nurse’s Health Study indicated that substitution of red meat with poultry, fish, nuts or legumes was associated with a lower risk of GDM in the US mothers with very higher red meat intake in comparison to the Iranian mothers^21^. Therefore, further research using innovative statistical methodologies such as substitution analysis are warranted to determine whether replacement of red and processed meat with other plant- and animal-based dietary protein sources is associated with a lower risk of GDM in Iranian pregnant women.

The interpretation of our findings has to take into account a number of limitations. Since our investigation was observational, residual confounding by unmeasured or unrecorded variables might skew the results. Second, the self-administered FFQ used to measure food intakes has the potential to misclassify research participants. Finally, because only the first trimester of pregnancy was included for our nutritional evaluation, any prospective changes in dietary intakes during pregnancy were not taken into account.

## Conclusion

This prospective birth cohort study indicated that consumption of red and processed meat during first trimester of pregnancy may be positively associated with the risk of GDM in a small sample of Iranian mothers, suggesting that intake of red and processed meat is associated with the risk of GDM, as found in Western countries, in a population with such a low red meat intake. Future cohort studies with larger sample sizes and more accurate dietary evaluations during pregnancy are warranted to examine this association, and to determine whether replacement of red and processed meat with other dietary protein sources is associated with a lower risk of GDM in Iranian pregnant mothers.

## Data Availability

The datasets used and/or analyzed during the current study will be available from the corresponding author on reasonable request.
